# Adeno-Associated Viral Vectors at the Frontier between Tolerance and Immunity

**DOI:** 10.3389/fimmu.2015.00120

**Published:** 2015-03-17

**Authors:** Federico Mingozzi, Hildegard Büning

**Affiliations:** ^1^Généthon, Evry, France; ^2^INSERM U974, Université Pierre et Marie Curie (Paris 6), Paris, France; ^3^Center for Molecular Medicine Cologne (CMMC), University of Cologne, Cologne, Germany; ^4^German Center for Infection Research (DZIF), Partner Site Bonn-Cologne, Bonn, Germany; ^5^Department I of Internal Medicine, University Hospital of Cologne, Cologne, Germany

**Keywords:** AAV vector, immune response, tolerance, vaccine, gene therapy

In recent years, the field of *in vivo* gene transfer with adeno-associated virus (AAV) vectors has seen an extraordinary expansion of applications and investments. Results emerging from clinical trials (1) and the recent market approval of a gene therapy drug for lipoprotein lipase deficiency (2) contributed to the hype around this vector system (3). Indeed, AAV vectors have several features that make them an ideal tool for gene transfer, for example, parental virions are replication deficient and non-pathogenic (4), and vectors can drive expression of a transgene for several years (5, 6) despite the fact that they do not integrate efficiently into the host genome. In recent years, a portfolio of natural AAV isolates (AAV serotypes) differing in tissue tropism has been developed as vectors. This toolbox has been further expanded with engineered AAV capsids developed to enhance efficiency and specificity of gene delivery, and to escape antibody neutralization (7). At the vector genome level, availability of potent promoter/enhancer sequences, codon-optimization of transgenes, and development of self-complementary AAV vectors (8) further enhanced efficacy of gene transfer. Finally, the availability of scalable processes to produce AAV vectors in GMP contributed significantly to the expansion of the field.

As the AAV vector technology reached a more mature stage, it has become clear that a better understanding of the interactions of viral vectors with the host immune system is needed. In this Research Topic of *Frontiers in Immunology*, the editors present a collection of reviews and research articles discussing the two sides of immune responses triggered by *in vivo* gene transfer. These responses in fact can be desirable when they result in induction of tolerance to the therapeutic transgene (9), or when they are exploited for vaccine development, as discussed by Nieto and Salvetti in their review article (10). Conversely, immunogenicity of the viral capsid or the transgene product can be detrimental, as it may result in lack or loss of efficacy following vector-mediated gene transfer.

Evidence for the critical role of tolerance induction in the achievement of sustained therapeutic efficacy following gene transfer comes from the work of Liao and colleagues, which provides evidence that glucocorticoid-induced TNF receptor (GITR) and its ligand GITR-L are of fundamental importance for the induction of immune regulatory responses in gene transfer and that lack of expression of GITR-L on antigen presenting cells results in impaired induction of regulatory T cells (Tregs) (11). Indeed, evidence of the key function of Tregs for successful *in vivo* gene therapy comes from several studies (12), and Liu and colleagues further demonstrate this concept in a model of plasmid gene transfer for hemophilia A, in which a combination of B cell depleting and Treg-enhancing drugs is used to successfully modulate transgene immunogenicity (13).

## Innate Immune Responses to AAV Vectors

The innate immune system constitutes the first line of defense against invading pathogens. It recognizes evolutionarily conserved structures foreign to the host or detects structures known as self, but present in the wrong intracellular compartment, via innate immune sensors termed pathogen recognition receptors (PRRs). Binding of such pathogen-associated molecular patterns (PAMPs) to PRRs activates the intracellular innate immune system, leading to substantial changes in the expression of genes related to host defense, in secretion of cytokines and chemokines, and up-regulation of co-stimulatory molecules, which as a consequence induce or modulate the adaptive arm of the immune system.

Of the four families of cellular PRRs [toll-like-receptors (TLRs), NOD-like receptors, RIG-like receptors, and C-type lectin receptors], as of now only two members of the TLR family, TLR-2 and TLR-9, have been described as sensors for AAV vectors. TLR-2 was identified as a PRR of the viral capsid in studies on cell autonomous immune responses in primary human liver cells (liver sinusoidal endothelial cells, Kupffer cells) and activated endothelial cells (14), while TLR-9 was reported as sensor of AAV vector genomes in plasmacytoid dendritic cells (pDC) isolated from mice and humans (15). Although both PRRs are part of the same family, recognition of the viral capsid caused induction of a Nuclear Factor kB-dependent inflammatory response (14), while activation of TLR-9 induced secretion of type I interferon (IFN) that was found to be enhanced if vectors with self-complementary (sc) AAV vector genomes were used (15, 16). The nature of this enhanced immunogenicity remains to be clarified, but is maybe related to a negative impact of sc vector genomes on capsid stability (16) or to the additional inverted terminal repeat (ITR) sequence present in sc vector genomes (8). The later hypothesis would be in line with a recent study reporting significantly reduced adaptive immune responses toward the capsid and the transgene product when using AAV vectors with a reduced number of CpG motifs, which are known TLR-9 PAMPs (17). The route of vector delivery appears to be a critical factor in AAV recognition by the innate immune system. The above-described activation of the TLR-9 myeloid differentiation primary response 88 (MyD88) signaling pathway, for example, resulted in humoral and T cell-mediated adaptive immune responses toward the AAV capsid and the transgene product in mice in which AAV vectors were administered intramuscularly. Conversely, following tail vein injection, neither a TLR-2, nor a TLR-9, or a type I IFN dependent induction of AAV specific IgG antibodies could be detected (15, 18).

## Adaptive Immune Responses in AAV Vector-Mediated Gene Transfer

Exposure to wild-type AAV or to AAV vectors, and the consequent activation of innate and adaptive immunity to vector and transgene leads to both antibody and cell-mediated responses. Antibodies directed against the AAV capsid are highly prevalent in humans (up to 60% of healthy individuals) and can efficiently neutralize the vector even when present at low titers, resulting in lack of efficacy, thus posing a significant constrain to patients enrollment in clinical trials. Similarly, vector administration results in long-lasting high-titer anti-AAV neutralizing antibodies (NAb), which prevent vector readministration. Results from human trials and studies conducted in small and large animal models of gene transfer showed that NAb titers as low as 1:5 can completely block AAV vector transduction, and that AAV vectors remain susceptible to antibody-mediated neutralization for several hours after intravascular delivery.

Two contributions on the topic of anti-AAV antibodies can be found in this Research Topic. Calcedo and Wilson reviewed the issue of NAb directed against AAV; in their manuscript, they discussed the prevalence of NAb in various human populations, the issue of antibody cross-reactivity, and finally the assays used to measure antibodies to AAV, and the strategies that could possibly be used to overcome this limitation (19). In the second review article, Tseng and Agbandje-McKenna (20) discuss different approaches to antibody epitope mapping and the relationship of these epitopes with the capsid structure. Furthermore, they suggest how this knowledge can be exploited to drive the efforts toward engineering novel AAV capsid variants resistant to antibodies, and to gain a better understanding on the structure-function-relationship across AAV serotypes when it comes to the interactions with the immune system.

In addition to neutralizing antibodies, natural infection with wild-type AAV also triggers cell-mediated immune responses against the capsid, which results in a reservoir of memory CD8^+^ T cells that can be reactivated upon vector administration. This can cause the destruction of transduced cells harboring AAV capsid antigen in the context of MHC class I, as it has been observed in subjects enrolled in AAV vector-mediated liver gene transfer trials. Several questions remain on the role of these capsid-specific CD8^+^ T cells in the outcome of gene transfer, as detection of T cell reactivity to the capsid in PBMCs has not always been associated with detrimental effects on gene transfer in liver and muscle trials. Notably, experience from the AAV8 gene therapy trials in hemophilia B subjects suggests that timely administration of immunosuppression can prevent detrimental effects of capsid-directed T cell immunity.

Three review articles in this Research Topic focus specifically on adaptive immune responses to AAV vectors in the context of gene transfer to different tissues, and discuss the issue of T cell-mediated immunity directed against the vector capsid. Willett and Bennett provide an overview of what it is known about gene transfer in an immune privileged body site, the eye, describing the unique and valuable lessons learned from the preclinical and clinical studies of AAV gene transfer for RPE65 deficiency (21). Ferreira and colleagues describe the experience with AAV vectors in muscle gene transfer in the context of the development of Glybera, the approved drug for the treatment of lipoprotein lipase deficiency (22). Finally, in their manuscript, Basner-Tschakarjan and Mingozzi provide a broad overview on the issue of T cell immunity to AAV vectors focusing on data emerging from gene therapy trials (23). To complete this collection of articles on immune responses in gene transfer, two review articles discuss the tools available to the investigators to study the immunogenicity of AAV vectors. Basner-Tschakarjan and colleagues provide an overview of *in vitro* and *in vivo* preclinical models that have helped to explain the immune responses to AAV vectors observed in human trials (24), while Britten and colleagues address the extremely important issue of immune assay standardization in clinical trials (25).

## Conflict of Interest Statement

The authors declare that the research was conducted in the absence of any commercial or financial relationships that could be construed as a potential conflict of interest.
